# Standardization and Development of Spray‐Dried Chewable Tablets From the Traditional Thai Remedy “Ya‐Tard Aob‐Choei”: HPLC Analysis and Comparison With Commercial Products

**DOI:** 10.1155/adpp/2822019

**Published:** 2026-10-19

**Authors:** Jringjai Areemit, Bungorn Sripanidkulchai, Sasipong Tipratchadaporn, Sarunya Tuntiyasawasdikul

**Affiliations:** ^1^ Center for Research and Development of Herbal Health Products Faculty of Pharmaceutical Science Khon Kaen University Khon Kaen 40002 Thailand kku.ac.th; ^2^ Phra Arjarn Fhan Arjaro Hospital Phanna Sub-District, Phanna Nikhom District Sakon Nakhon 47130 Thailand

**Keywords:** chewable tablets, HPLC standardization, phytochemical analysis, spray drying, Thai traditional medicine, Ya-Tard Aob-Choei

## Abstract

**Ethnopharmacological Relevance:**

The Thai traditional medicine “Ya‐Tard Aob‐Choei” (AC) is a polyherbal remedy used for the treatment of gastrointestinal disorders. Its traditional liquid dosage form has practical limitations, including inconsistent appearance and difficulties in handling and transportation. This study aimed to develop a chewable dosage form of AC with satisfactory physicochemical properties and to evaluate the retention of selected marker compounds, including gallic acid (GA), cinnamaldehyde (CMD), eugenol (EUG), and glycyrrhizic acid (GZ).

**Materials and Methods:**

Formulation studies were conducted to identify suitable excipients for the preparation of AC powder and chewable tablets, and the resulting formulations were evaluated for their physicochemical properties. A validated high‐performance liquid chromatography (HPLC) method was used to determine the selected marker compounds and compare the phytochemical profile of the developed formulation with liquid AC preparations, including a self‐prepared reference formulation and commercially available products.

**Results:**

The formulation containing maltodextrin DE 18 at an extract‐to‐carrier ratio of 1:2 exhibited optimal powder characteristics for tablet production, and isomalt was identified as an effective diluent–binder for the preparation of chewable tablets with satisfactory physicochemical properties. HPLC analysis revealed compound‐specific changes during conversion from the liquid‐to‐solid dosage form. GA was relatively well preserved (5.25 mg/dose), whereas EUG, CMD, and GZ decreased by approximately 99%, 35%, and 33%, respectively, compared with the self‐prepared liquid formulation (P7).

**Conclusion:**

A chewable AC tablet with satisfactory physicochemical properties was developed; however, conversion to the solid dosage form resulted in compound‐specific losses, particularly of EUG. Further process optimization is required to improve the retention of volatile and heat‐sensitive constituents. This formulation approach may nevertheless offer a pragmatic strategy for the development and standardization of other traditional Thai herbal medicines, although dedicated stability and sensory studies are needed to establish product stability and acceptability.

**Highlight**
• A traditional liquid Thai herbal medicine was developed as a chewable tablet.
• Selected phytochemical markers were quantified using a validated high‐performance liquid chromatography (HPLC) method.
• Conversion to a solid dosage formulation resulted in compound‐specific changes, with substantial loss of volatile eugenol (EUG).
• Spray drying produced powders with suitable properties for tablet formulation.
• This approach may support development and standardization of other Thai herbal medicines.

## 1. Introduction

Recently, the use of Thai traditional medicines has been increasing worldwide. Thai people have traditionally used herbal medicines to treat a variety of symptoms and ailments, as well as for health promotion. The cinnamon stomachic mixture known in Thai as “Ya‐Tard Aob‐Choei” (AC) is one of the traditional medicine formulations listed in the National List of Essential Medicines in Thailand [1] and is commonly used as a carminative in general hospitals to alleviate symptoms of bloating, abdominal distention, and flatulence. The AC recipe is composed of five herbal materials, namely, *Cinnamomum zeylanicum* Blume, *C. bejolghota* Wight, *Amomum kravanh* Pierre ex Gagnep, *Syzygium aromaticum* (L.) Merril & Perry, and *Glycyrrhiza glabra* L., at an equal ratio of 1:1:1:1:1 (w/w), with 800 mg of each herbal material per 100 mL of the formulation, as shown in Figure 1. Four compounds, namely, gallic acid (GA), cinnamaldehyde (CMD), EUG, and glycyrrhizic acid (GZ), were selected as representative quality control markers based on their characteristic occurrence in the principal herbal ingredients of the AC formulation and their relevance to the chemical characterization of these herbal materials. GA is a characteristic phenolic compound reported in *S*. *aromaticum*, CMD is a major constituent of *C. zeylanicum*, EUG is a characteristic constituent of both *S. aromaticum* and *C. zeylanicum*, and GZ is a major characteristic constituent of *G. glabra* [2–6]. These compounds have been used as analytical markers for the characterization and quality control of the corresponding herbal materials and preparations. Therefore, GA, CMD, EUG, and GZ were selected as representative chemical markers for simultaneous HPLC analysis to support the quality control and standardization of the AC formulation.

**FIGURE 1:**
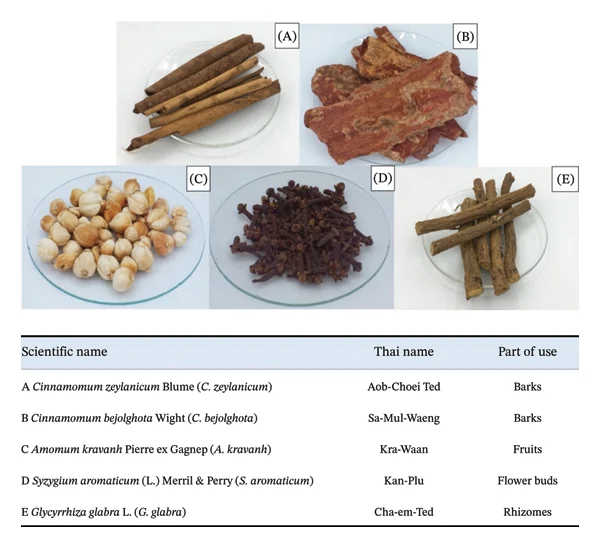
Scientific names, Thai names, and plant parts of the herbal materials used in the AC formulation.

The AC recipe was prepared by heating the mixture of herbs in water at 85°C–90°C and filtering to get a clear solution. A previous study showed that the AC recipe had similar efficacy and safety to simethicone for the treatment of patients with functional dyspepsia [7]. Commercial AC products are available in liquid dosage forms, which may show batch‐to‐batch color inconsistency and lower stability of active ingredients. The use of plastic or glass containers for the AC recipe may present challenges during transportation and storage, including potential bottle inflation or leakage due to gas production within the packaging, handling difficulties, and microbiological concerns. To overcome these limitations, a solid dosage form of the AC recipe is an alternative because it is relatively easy to manufacture and may provide good physical and chemical stability.

Chewable tablets are an oral dosage form intended to be chewed to facilitate the release of active ingredients and may be a suitable dosage form for the development of the AC recipe because of their ease of administration, portability, and the fact that they do not require water for administration [8]. To develop a chewable tablet from the AC recipe, the aqueous extract of the five herbal materials needs to be converted into a solid form suitable for incorporation into the tablet formulation. Spray drying is considered a relatively simple and rapid process for producing dry herbal extracts and has potential for scale‐up [9–11]. Maltodextrin (MD), a polysaccharide produced by the partial enzymatic hydrolysis of starch, is a commonly used carrier because of its high‐water solubility, low cost, and availability [12, 13]. Despite the established use of the AC recipe as a traditional liquid preparation, research on its pharmaceutical development remains limited. Previous studies have mainly focused on the clinical efficacy and traditional use of the liquid formulation, whereas systematic development of the AC recipe into a solid oral dosage form has not been adequately investigated. In particular, limited evidence is available regarding the feasibility of converting the aqueous AC preparation into a spray‐dried powder suitable for incorporation into a chewable tablet and the extent to which the formulation process affects the content of characteristic marker compounds. Furthermore, comparative information on the marker‐compound profiles of the developed solid formulation, the original liquid AC preparation, and commercially available AC products remains limited. Addressing these research gaps is important for the quality control and standardization of a solid dosage form derived from a traditional herbal preparation.

Therefore, the aims of this study were to develop a chewable tablet containing the AC recipe, evaluate its physicochemical properties, and develop and validate a simultaneous HPLC method for the identification and quantification of four characteristic marker compounds. The marker‐compound profiles of the developed chewable tablets were further compared with those of the original liquid AC preparation and commercially available AC products in Thailand.

## 2. Materials and Methods

### 2.1. Materials

Dried barks of *C. zeylanicum* (Voucher Specimen No. HB215/69), dried barks of *C. bejolghota* (voucher specimen No. HB217/69), dried fruits of *A. kravanh* (voucher specimen No. HB218/69), dried flower buds of *S. aromaticum* (voucher specimen No. HB219/69), and dried rhizomes of *G. glabra* (voucher specimen No. HB216/69) were obtained from Fan Arjaro Hospital, Sakon Nakhon Province, Thailand. The plant materials were authenticated by a qualified botanist, and the corresponding voucher specimens were deposited at the Center for Research and Development of Herbal Health Products, Khon Kaen University, Thailand. After receipt, the herbal materials were stored in well‐closed containers at room temperature and protected from light and moisture until use. Microcrystalline cellulose (Avicel pH‐102; FMC Biopolymer, Ireland), MD dextrose equivalent (DE) 10 and MD DE 18 (MD; Neo‐Maldex NM‐10, Neotech Food Co., Ltd, Thailand), lactose monohydrate (Kerry Ingredients India Private Limited, India), corn starch (Hubei Ipure Import & Export, China), polyvinyl pyrrolidone (PVP K30 and K90, Nanhang Industrial Co., Ltd, China), colloidal silicon dioxide (Aeropearl 300 Pharma, Evonik, Singapore), talcum (Haicheng Xinnuoxier Minning Industry Co., Ltd, China), and magnesium stearate (Faci Asia Pacific Pte Ltd., Singapore) were used as excipients. GA, GZ, and EUG reference standards were purchased from Sigma‐Aldrich (USA), while CMD was obtained from ChemFaces (China). The purity and lot number of each reference standard were as follows: GA (97.5%, Lot No. 023K0117), GZ (98%, Lot No. 17052701), CMD (98%, Lot No. CFS202301), and EUG (99%, Lot No. MKBH6197V). HPLC grade acetonitrile (HPLC grade, Labscan, Bangkok, Thailand) and methanol (HPLC grade, Fisher Scientific, Loughborough, UK) were used. All of the chemical reagents used were of analytical grade.

### 2.2. Validation of HPLC Method for Determination of Major Compounds

The HPLC assay to analyze four active compounds in the AC recipe was validated in terms of sensitivity, linearity, accuracy, and precision [14]. The compounds were determined using a reversed‐phase HPLC system (Agilent 1200 series, Germany) equipped with a quaternary pump and a 1260 Infinity VWD detector, with a Hypersil gold C18 column (4.6 × 150 mm). Detection was performed at 254 nm for GZ and at 280 nm for GA, CMD, and EUG. The column temperature was set at 30°C. The mobile phase consisted of 0.5% (v/v) acetic acid in acetonitrile (A) and 0.5% (v/v) acetic acid in water (B), delivered according to the gradient program shown in Table 1. The flow rate was 0.8 mL/min, the injection volume was 20 µL, and the column temperature was maintained at 30°C. The total analysis time was 68 min, followed by a column equilibration period of 2 min before the subsequent injection.

**TABLE 1:** Gradient elution program for simultaneous HPLC analysis of GA, CMD, EUG, and GZ.

Time (min)	0.5% acetic acid in acetonitrile (%)	0.5% acetic acid in water (%)
0	0	100
5	10	90
15	20	80
20	20	80
25	30	70
35	40	60
37	40	60
45	60	40
60	10	90
65	0	100
68	0	100

### 2.3. The Preparation of AC Recipe Spray‐Dried (AC‐SD) Powder

#### 2.3.1. Preparation of AC Recipe Suspensions

The quality of all herbs in the recipe was evaluated in terms of total ash, acid‐insoluble ash (%), and heavy metals before use. The AC recipe was prepared according to the standard preparation process from Fan Arjaro Hospital (Sakon Nakhon Province, Thailand) with some modifications. In brief, all dried herbs were ground and screened with a mesh sieve No. 120 before use. The dry powder of herbs was added to hot water in a weight ratio of 4 to 100 at 85°C–90°C and stirred at 30‐min intervals for 6 h. Then, it was allowed to cool at room temperature and filtered by using cellulose filter paper with a pore size of 11 μm. The herbal extract was kept at 8°C–10°C until use. Before use, the herbal extract was removed from cold storage and allowed to reach room temperature.

#### 2.3.2. Determination of Solid Content in AC Recipe Suspensions

A specified volume of the AC recipe suspension (V_0_) was frozen and dried at −80°C under 0.100 mbar using a freeze dryer (Christ, Germany). The percentage solid content was calculated from the weight of solid remaining after freeze drying (W) as shown in Equation (1).

(1) Solid content%w/v=W×100V0.

#### 2.3.3. Preparation of AC‐SD Powder

The AC recipe suspension was mixed with MD at AC‐solid‐to‐MD ratios of 1:1 and 1:2. The resulting solutions were then dried using a pressure‐nozzle type spray dryer (GEA; Germany). During preliminary method development, spray drying conditions were explored over the following ranges: inlet temperature 120°C–180°C, outlet temperature 65°C–85°C (dependent variable), aspirator efficiency 100%, and feed flow rate 10 mL/min. For the tablet‐formulation batch (MD‐18–12), the following parameters were applied: inlet temperature, 120°C; outlet temperature, 65°C–85°C (recorded range); aspirator setting, 100%; and feed rate, 10 mL/min. It should be noted that these parameters were selected based on preliminary feasibility screening and that further systematic optimization of spray drying conditions is warranted to improve the retention of thermolabile compounds. The AC‐SD was kept in an air‐tight container at controlled room temperature (25 ± 2°C/50 ± 5% RH) and protected from light and moisture until use.

#### 2.3.4. Characterization of AC‐SD

The morphology of the powder was investigated using scanning electron microscopy (SEM). The sample was mounted on an aluminum stub using double‐sided adhesive tape and then coated with a gold palladium alloy and examined at 1000x magnification. The moisture content of AC‐SD was determined by accurately weighing 3.0 g of sample into an aluminum weighing pan and heating it at 105°C by using a halogen moisture analyzer (Mettler Toledo HE53, Switzerland). Measurements were done in triplicate.

For micromeritic properties, the particle size distribution of AC‐SD was determined by the dry method using a particle size analyzer (Malvern Instrument, UK) under the following conditions: air pressure 2.0 bar, feed rate 3%, obscuration 2%–6%, measurement time 3.5 s, and measurement cycle 3 per aliquot. Measurements were done in 10 cycles. The results are expressed as mean ± SD. Bulk and tapped densities of AC‐SD were evaluated by accurately weighing 20 g of sample (W_1_) into a 100‐mL cylinder without compaction. The unsettled apparent volume (V_0_) was read to the nearest graduated unit before the cylinder was tapped 500 and 1250 times from an approximate height of 3.0 mm. The corresponding settled volume was read to the nearest graduated unit and recorded as V_500_ and V_1,250_. If the difference between V_500_ and V_1,250_ was less than or equal to 1 mL, V_1,250_ was recorded as the final tapped volume (V_F_). If the difference between V_500_ and V_1,250_ exceeded 1 mL, the tapping was repeated in increments of 1250 taps until the difference between succeeding measurements was less than or equal to 1 mL. The untapped bulk density (g/mL) was calculated using Equation (2), and the tapped density (g/mL) was calculated using Equation (3). The compressibility index (CI) was also measured to determine the propensity of a powder to be compressed relative to its flow. CI was calculated using Equation (4) [15]. All parameters are expressed as mean ± SD.

(2) Bulk densityg/mL=W1V0,

(3) Tapped densityg/mL=W1VF,

(4) Compressibility index%=V0−VF×100V0.

### 2.4. Formulation Development of Chewable Tablets of AC‐SD

The chewable tablets were prepared according to the direct compression method and the wet granulation method. For the direct compression method, all ingredients were mixed homogeneously and compressed with a rotary tableting machine (U.D. Machinery, Thailand) with 15‐mm flat and round punches and dies. For the wet granulation method, AC‐SD, diluents, and binder were blended homogeneously in a mortar and pestle. Isopropyl alcohol was gradually added into the mixture during blending until a damp mass formed. The damp mass was then passed through a mesh sieve No. 12 to get wet granules. The granules were dried at 50°C in a tray dryer (Charatchai Machinery, Thailand) until the moisture content was not more than 3.0%. Dried granules were screened using a mesh sieve No. 30 and mixed with the rest of the excipients in the formulation. Tablets were compressed with the same equipment as described above.

### 2.5. Characterization of Chewable Tablets

For the present proof‐of‐concept study, each tablet formulation (F1–F4) was prepared as a single batch. From each formulation, certain tablets were randomly sampled for physicochemical and analytical testing.

#### 2.5.1. Weight Variation

Twenty whole tablets were weighed individually using an analytical balance (Mettler Toledo, USA), and the average weight was calculated. The requirements were met if the weights of at most two of the tablets deviated from the average weight by more than 5% and no single tablet deviated by more than 10%. Then, mean ± SD was calculated. Content uniformity of individual tablets was not determined in this proof‐of‐concept study.

#### 2.5.2. Thickness and Hardness

The thickness and hardness of 10 tablets were measured individually using a thickness–hardness tester (Erweka, Germany). Then, mean ± SD was calculated.

#### 2.5.3. Friability

Ten whole tablets were carefully dedusted before testing. The tablet sample was accurately weighed (W_1_) and placed in the drum of a friability tester (Erweka, Germany). The drum was rotated at 25 rpm for 100 revolutions. The tablets were removed, dedusted as before, and accurately weighed (W_2_). The percentage of friability was calculated using Equation (5). The acceptance criterion was friability of not more than 1.0%.

(5) Friability%=W1−W2×100W1.

#### 2.5.4. Disintegration Time

Disintegration time was determined using a disintegration tester (Electrolab, India). Briefly, one dosage unit was placed in each of the six tubes of the basket, and a disc was added. The apparatus was operated using water as the immersion fluid, maintained at 37 ± 2°C. The time required for complete disintegration of each tablet was recorded. The disintegration times of six individual tablets were recorded, and the results are reported as a minimum–maximum range in minutes:seconds (min:sec). In vitro dissolution or drug release testing of the individual marker compounds was not performed in the present study. Such compound‐specific dissolution/release testing should be included in subsequent formulation development to further evaluate the release performance of the chewable tablet.

### 2.6. Comparison of the Main Components in Chewable Tablets and AC Recipe Products

The chewable tablets of AC‐SD were ground and accurately weighed. The powder was dissolved in 5 mL of methanol. Each sample was vortexed, sonicated for 10 min, and then filtered through a 0.45‐μm‐nylon syringe filter. The estimated average active content of each marker compound per tablet was determined using the HPLC method described in Section 2.2.

The commercial products of AC recipe were purchased from local drug stores and hospitals in Thailand. All the samples were in liquid form. The samples underwent filtration using a 0.45‐μm‐nylon syringe filter. The quantification of the active substances in each sample was performed using the HPLC method described in Section 2.2.

### 2.7. Statistical Analysis

All quantitative data are presented as mean ± standard deviation (SD). Statistical differences among multiple groups were analyzed using one‐way analysis of variance (ANOVA). Differences were considered statistically significant at *p* < 0.05 (95% confidence level). All statistical analyses were performed using IBM SPSS Statistics for Windows, Version 28.0.1 (IBM Corp., Armonk, NY, USA).

## 3. Results and Discussion

### 3.1. Validation of HPLC Method for Determination of Major Compounds

The HPLC assay demonstrated good linearity, precision, and high accuracy for the four major compounds GA, CMD, EUG, and GZ (Table 2). The HPLC chromatograms of the standards and AC recipe are shown in Figure 2A,B. The retention times of GA, CMD, GZ, and EUG were 11, 35, 40.5, and 41 min, respectively. Linear regression of peak area versus concentration showed good linear relationships with *R* ^2^ values greater than 0.999 (linear range: 5–50 µg/mL). The limit of detection (LOD) and limit of quantitation (LOQ) values for the major compounds ranged from 0.43 to 2.18 and from 1.44 to 7.28 µg/mL, respectively, indicating the sensitivity of the proposed method. Accuracy was reported as percent recovery following assay of known amounts of analyte added to the sample at three concentrations of each major compound (5, 25, and 50 µg/mL). The results showed that the percent recovery of the major compounds ranged from 96.25% to 103.72% across the tested concentrations (Table 2), indicating acceptable accuracy of the proposed method [16]. Intraday and interday precision were expressed as relative standard deviation (RSD) values in the range of 0.65% to 1.09% and 0.35% to 1.97%, respectively. These values were in the acceptable range [17].

**TABLE 2:** HPLC method validation parameters for the determination of major compounds in AC recipe.

Major compounds	Linearity (*R* ^2^)	LOD (µg/mL)	LOQ (µg/mL)	Accuracy (% recovery)			Precision (% RSD)					
				Concentration (µg/mL)			Intraday (µg/mL)			Interday (µg/mL)		
				5	25	50	5	25	50	5	25	50
GA	1.0000	0.43	1.44	101.92 ± 1.15	101.10 ± 0.40	100.49 ± 0.94	0.88	0.93	1.03	1.13	0.36	0.66
CMD	0.9999	0.63	2.09	102.20 ± 0.81	101.32 ± 0.51	100.02 ± 1.05	0.77	0.74	0.76	1.97	0.45	0.94
GZ	0.9993	2.18	7.28	96.25 ± 0.90	98.80 ± 0.39	100.41 ± 1.02	0.71	0.84	1.09	1.65	0.35	0.91
EUG	0.9999	0.68	2.27	103.72 ± 1.34	101.54 ± 0.93	99.24 ± 0.87	0.65	0.83	0.78	1.87	0.42	1.03

**FIGURE 2:**
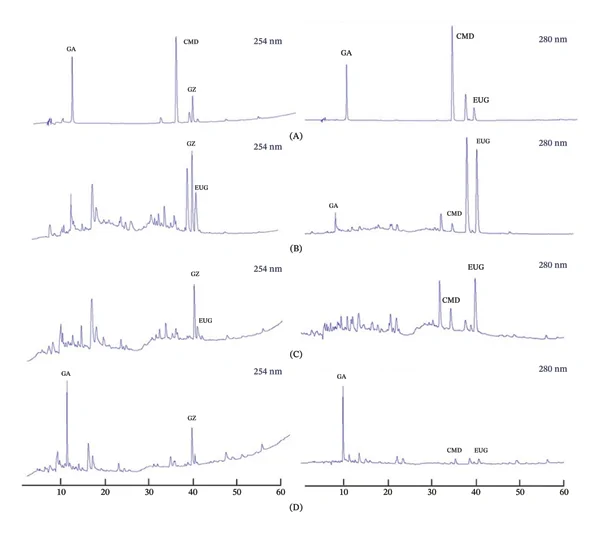
HPLC chromatograms of the reference standards of gallic acid (GA), cinnamaldehyde (CMD), glycyrrhizic acid (GZ), and eugenol (EUG) (A); P7 AC recipe suspension prepared in‐house according to the AC recipe (B); AC‐SD (C); AC‐SD chewable tablets (D), detected at 254 and 280 nm.

### 3.2. The Preparation of Spray‐Dried Powder of AC Recipe (AC‐SD)

#### 3.2.1. Contaminants of Herbal Materials

Quality control of herbal materials should be conducted to ensure their purity, cleanness, and safety before use in the development process because they originate from nature. Total ash is used as an indicator to evaluate the purity and cleanliness of herbal materials (e.g., sand and soil), adhering to the plant surface. Acid‐insoluble ash is used to determine the amount of nonvolatile agents such as sand and siliceous earth [18]. It was found that the amounts of total ash and acid‐insoluble ash of *C. zeylanicum*, *A. kravanh, S. aromaticum,* and *G. glabra* were within the acceptance limits, as shown in Table 3, meeting the acceptance criteria used in this study [19–22]. Although no textual references specifying acceptance limits for total ash and acid‐insoluble ash were available for *C. bejolghota*, the obtained values were considered acceptable based on their relatively low levels. However, the total ash content of *A. kravanh* was 9.98%, which was close to the specified limit of NMT 10.0%, leaving a narrow margin of 0.02 percentage points. This narrow margin may increase the risk of specification failure due to normal batch‐to‐batch variability; therefore, total ash should be closely monitored in subsequent batches to ensure consistent compliance with the specification.

**TABLE 3:** Particle size distribution and physicochemical properties of maltodextrin and AC‐SD.

Samples	Particle size (micron)			Properties			
	D10	D50	D90	Bulk density (g/mL)	Tapped density (g/mL)	Compressibility index (%)	Moisture content (%)
MD‐10	13.83 ± 0.02^a^	35.37 ± 0.11^a^	66.33 ± 0.28^a^	0.27 ± 0.04^a^	0.42 ± 0.02^a^	34.40 ± 0.01^a^	1.22 ± 0.04^a^
MD‐18	15.87 ± 0.20^b^	44.08 ± 0.73^b^	86.05 ± 1.56^b^	0.29 ± 0.06^a^	0.43 ± 0.06^a^	34.59 ± 0.02^a^	1.05 ± 0.04^b^
MD‐10‐11	2.70 ± 0.02^c^	7.02 ± 0.01^c^	15.09 ± 0.03^c^	0.19 ± 0.01^b^	0.41 ± 0.03^a^	52.97 ± 1.48^b^	5.95 ± 0.12^c^
MD‐10‐12	3.04 ± 0.04^d^	8.08 ± 0.03^d^	16.26 ± 0.02^d^	0.27 ± 0.01^a^	0.41 ± 0.02^a^	34.77 ± 2.10^a^	5.62 ± 0.14^d^
MD‐18‐11	4.05 ± 0.01^e^	10.57 ± 0.03^e^	21.84 ± 0.15^e^	0.25 ± 0.02^a^	0.49 ± 0.02^b^	48.98 ± 1.27^c^	6.07 ± 0.16^c^
MD‐18‐12	4.48 ± 0.02^f^	11.97 ± 0.03^f^	23.78 ± 0.19^f^	0.33 ± 0.00^c^	0.42 ± 0.01^a^	21.38 ± 1.95^d^	5.02 ± 0.06^e^

*Note:* Values are presented as mean ± SD. Different superscript letters within the same column indicate statistically significant differences among groups (one‐way ANOVA, *p* < 0.05).

In terms of safety, contamination with toxic heavy metals is an important concern. Cultivation, harvesting, and storage can result in heavy metal residues in herbal materials because of contamination of water and soil. Heavy metal contamination is an important factor related to the toxicity of herbal preparations [23–25]. Thus, residual heavy metals should be strictly controlled. This study revealed that the herbal materials examined met the acceptability requirements for the presence of arsenic, lead, and cadmium, as indicated in Table 4. The acceptance criteria for toxic elements have been determined according to the acceptable daily intake (ADI) values. These values are set at a maximum of 4 parts per million (ppm) for arsenic, a maximum of 10 ppm for lead, and a maximum of 0.3 ppm for cadmium [26].

**TABLE 4:** Total ash, acid‐insoluble ash, LOD, and heavy metal content and acceptance criteria of herbal materials.

Type of herbs	Total ash (%)	Acceptance criteria	Acid‐insoluble ash (%)	Acceptance criteria	LOD (%)	Acceptance criteria	Arsenic (ppm)	Lead (ppm)	Cadmium (ppm)
*C. zeylanicum*	4.22 ± 0.07	NMT 6.0%	0.17 ± 0.05	NMT 4.0%	6.16 ± 1.68	—	1.21 ± 0.12	0.11 ± 0.03	ND
*C. bejolghota*	3.08 ± 0.04	—	0.08 ± 0.01	—	4.66 ± 0.03	—	1.17 ± 0.06	0.42 ± 0.02	ND
*A. kravanh*	9.98 ± 0.16	NMT 10.0%	0.13 ± 0.01	NMT 3.0%	7.90 ± 0.12	NMT 11%	0.30 ± 0.06	0.13 ± 0.02	ND
*S. aromaticum*	5.40 ± 0.06	NMT 7.0%	0.10 ± 0.04	NMT 0.5%	10.20 ± 0.04	NMT 12%	1.11 ± 0.05	0.09 ± 0.02	ND
*G. glabra*	5.43 ± 0.01	NMT 7.0%	0.05 ± 0.05	NMT 2.0%	4.24 ± 0.14	—	0.93 ± 0.12	0.23 ± 0.01	ND

*Note:* Values are presented as mean ± SD. ND: not detected.

#### 3.2.2. Determination of Solid Content in AC Recipe

To determine the quantity of solid‐form AC equivalent to the conventional dosage regimen for incorporation into the tablet formulation, the solid content was determined using a freeze‐drying method. The solid content of the AC recipe was 0.83%, indicating that 100 mL of the liquid AC recipe contained 0.83 g of solid content. The conventional oral dosage regimen of the AC recipe is 15–30 mL, three times daily, after meals. In this study, the maximum single dose of 30 mL was used to calculate the amount of solid‐form AC required per tablet. Accordingly, each tablet was designed to contain 0.25 g of solid‐form AC. The final F4 chewable tablet had a total weight of 1.00 g and contained 0.1% w/w camphor, corresponding to 1.0 mg of camphor per tablet. At the intended dosage of one tablet three times daily, the maximum daily camphor exposure was 3.0 mg/day.

#### 3.2.3. Preparation and Characterization of AC‐SD Powder

Several methods, such as lyophilization or freeze drying, can be employed to achieve a solid state in a sample. Freeze drying is a method that involves removing water from a frozen substance by sublimation under reduced pressure. This process is both time‐consuming and costly, and it can only be used for dry, heat‐sensitive, and high‐value products [27–30]. The spray drying process is considered a simpler and faster process. It uses atomization to form microdispersed droplets. Then, the water in these droplets quickly evaporates when passed through a stream of hot air inside the chamber, resulting in dry particles collected by a cyclone. This technique is cost‐effective, can be operated continuously, and has the potential for scale‐up [9–11]. It is well established in the literature that the glass transition temperature (Tg) plays a critical role in the stability of spray‐dried products. When the storage or processing temperature exceeds Tg, the material transitions from a rigid, glassy state to a soft, viscoelastic state, leading to stickiness, agglomeration, and caking [31, 32]. The addition of high‐molecular‐weight carriers such as MD is a widely documented strategy to increase the Tg of the system, thereby reducing these undesirable effects [33, 34]. Based on this established literature rationale, MD was selected as the carrier for the present study. However, Tg values were not directly measured by DSC or other thermal analysis techniques in this work; the selection of the optimal formulation (MD‐18–12) was therefore based on the experimentally determined powder characteristics described below. MD, a polysaccharide derived from starch through partial enzymatic hydrolysis, is a particularly interesting choice of carrier due to its high solubility in water, natural origin, and low cost.

In this study, two MDs with different DE values, MD DE 10 (MD‐10) and MD DE 18 (MD‐18), were used as carriers for AC in the spray drying process under the same conditions. The weight ratio of solid content of AC to MD was 1:1 and 1:2. The morphology of AC‐SD (MD‐10‐11, MD‐10‐12, MD‐18‐11, and MD‐18‐12) exhibited a spherical shape with a more wrinkled surface compared with spray‐dried MD‐10 (MD‐10 (SD)) and MD‐18 (MD‐18 (SD)) as shown in Figure 3. This phenomenon can be due to many factors, such as heat transfer from the surface to the deepest point inside the droplet, drying residence time, drying temperature, and drying rate [35]. In addition, droplet size distribution is one of the most impactful factors on the particle morphologies produced after the drying process. Larger particles (20–100 μm) were generally more wrinkled, while small particles (5–20 μm) were often classified as hollow [36, 37]. However, all AC‐SD particles were spherical and separated from each other as shown in particle size distribution data (Table 3), making them suitable for the production of mixed medication powder for tablet compression. It should be noted that the D50 values of all AC‐SD samples (7.02–11.97 µm) were substantially smaller than those of spray‐dried MD alone (MD‐10: 35.37; MD‐18: 44.08 µm), despite the use of the same nominal spray‐drying conditions. This difference may be associated with differences in feed composition and, consequently, feed properties such as solids content and viscosity. The AC‐SD feeds contained the herbal extract together with MD, while the contribution of the herbal extract to the total solids was relatively low (0.83% w/v). Such differences in feed properties may affect atomization and droplet formation, resulting in differences in the particle size of the dried powders. In particular, lower feed viscosity may facilitate the formation of smaller droplets during atomization and consequently produce finer particles. However, because feed viscosity and surface tension were not directly measured in the present study, these factors are considered possible contributors rather than experimentally confirmed mechanisms. The bulk density of AC‐SD increased as the MD ratio increased from 1:1 to 1:2 for both MD DE values. Among AC‐SD samples, MD‐18–12 exhibited the highest bulk density, indicating that MD‐18–12 was denser and flowed better than the others. This was confirmed by the CI values, which showed that the flowability of AC‐SD followed the order MD‐18‐12 > MD‐10‐12 > MD‐18‐11 > MD‐10‐11 as shown in Table 4. Interestingly, the moisture content of AC‐SD tended to decrease as the MD ratio increased for both DE types. However, the effect of DE on moisture content was ratio dependent. At the 1:2 ratio, MD‐18‐12 showed a lower moisture content (5.02%) than MD‐10–12 (5.62%), whereas at the 1:1 ratio, MD‐18‐11 (6.07%) exhibited a slightly higher moisture content than MD‐10‐11 (5.95%). Thus, increasing the DE of MD did not consistently reduce moisture content across the tested MD ratios. Nevertheless, the lower moisture content, together with the superior bulk density and flowability of MD‐18‐12, supported its selection as the optimal AC‐SD for tablet‐formulation development. Future studies should include DSC analysis to determine the Tg of the selected spray‐dried powder and experimentally evaluate the role of MD in improving its thermal stability.

**FIGURE 3:**
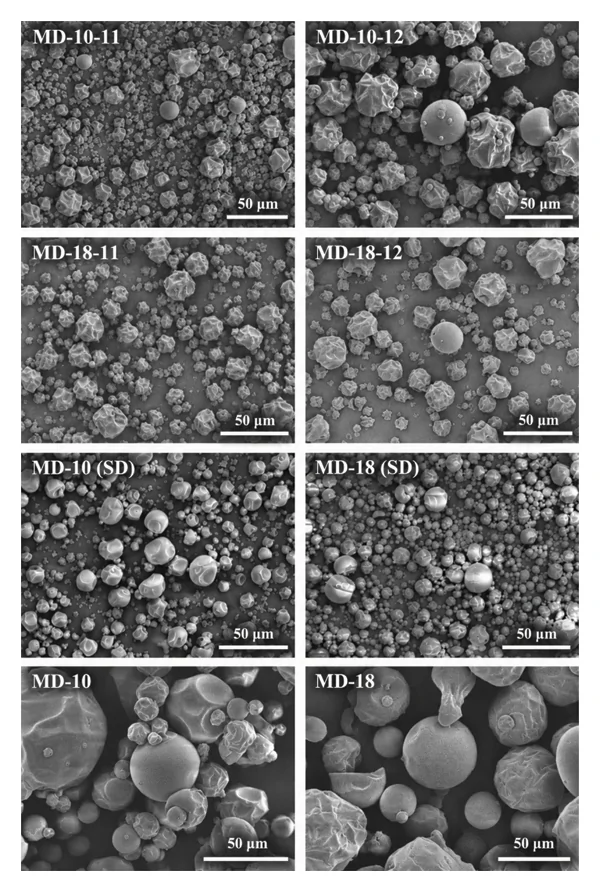
Scanning electron microscope (SEM) images of AC‐SD with MD‐10 in 1:1 weight ratio (MD‐10‐11), AC‐SD with MD‐10 in 1:2 weight ratio (MD‐10‐12), AC‐SD with MD‐18 in 1:1 weight ratio (MD‐18‐11), AC‐SD with MD‐18 in 1:2 weight ratio (MD‐18‐12), spray‐dried maltodextrin DE‐10 (MD‐10 [SD]), spray‐dried maltodextrin DE‐18 (MD‐18 [SD]).

### 3.3. Formulation Development and Characterization of Chewable Tablets

Based on the traditional AC recipe, the solid form of AC should be 0.25 g per tablet. In this study, we decided to use AC‐SD with MD‐18 in 1:2 weight ratio. Thus, the quantity of MD‐18‐12 in one tablet should be 0.75 g. This will allow the patient to follow the same dosing regimen of AC formula in liquid form: one tablet three times daily after meals.

Four formulations of chewable tablets were created, as depicted in Table 5. PVP K30 was selected as the binder, while a mixture of lactose monohydrate and maize starch was chosen as the diluent (F1–F2). The flowability of F1 was good, but powder spilled from the die cavity during compression, leading to a significant weight variation and limiting the control of the target weight. This might be a result of the low bulk density of the formulation, which includes up to 75% MD‐18–12, leading to a high requirement for the die cavity. An attempt was made to address this problem by increasing PVP K30 from 0.5% to 3.0% w/w in F2. However, the result was similar to that obtained with F1, leading to the replacement of PVP K30 with PVP K90. Formulation (F3) contained 3% w/w of PVP K90, and the tablets were successfully produced without weight‐variation problems. This may be attributed to the higher molecular weight of PVP K90. PVP K30 has a molecular weight of 50,000 Da, whereas PVP K90 has a molecular weight of 1,000,000 Da. The elevated molecular weight of PVP K90 leads to increased adhesive properties of the powder blend [38]. As a result, the granules became denser with a Carr’s index value within the range of excellent flowability, allowing the die cavity to be filled consistently and the tablets to meet the weight‐variation requirements. Although the friability was considerable, it remained within the specified limit, while the hardness met the acceptance criteria. Isomalt was used as a diluent instead of the lactose monohydrate and corn starch mixture (F4) to reduce tablet friability. The results indicated a significant decrease in friability together with acceptable tablet properties, as demonstrated in Table 6 and Figure 4C. This could be due to the advantageous properties of isomalt, which may enhance the binding capabilities of PVP K90, resulting in a synergistic effect. Isomalt is typically utilized as a diluent and sweetening agent in tablet formulations. Previous studies have shown that isomalt can be used as a filler‐binder in directly compressed tablets without adding other binding agents [39, 40]. Isomalt was found to be suitable as a filler‐binder in wet granulation compressed tablets with a high concentration of natural extract in this investigation.

**TABLE 5:** Formulation of chewable tablets.

Ingredients	Function	F1	F2	F3	F4
MD‐18–12	API	75	75	75	75
Lactose monohydrate	Diluent	11.5	13.5	10.5	—
Corn starch	Diluent	9.5	5.0	8.0	—
Isomalt	Diluent	—	—	—	19.09
Mannitol	Diluent	—	—	—	—
PVP K30	Binder	0.5	3.0	—	—
PVP K90	Binder	—	—	3.0	3.0
Isopropyl alcohol	Solvent	qs.	qs.	qs.	qs.
Menthol	Flavoring agent	0.5	0.5	0.5	0.3
Camphor	Flavoring agent	0.5	0.5	0.5	0.1
Colloidal silicon dioxide	Glidant	1	1	1	1
Sucralose	Sweetener	—	—	—	0.01
Talcum	Glidant	1	1	1	1
Magnesium stearate	Lubricant	0.5	0.5	0.5	0.5
Total		100	100	100	100

**TABLE 6:** Physicochemical properties of final blend powder and tablets.

Properties	F1	F2	F3	F4	Acceptance criteria
Bulk density of final blend (g/mL)	0.40	0.42	0.39	0.38	—
Tapped density of final blend (g/mL)	0.45	0.46	0.44	0.48	—
Compressibility index of final blend (%)	12.01	9.05	11.36	20.83	—
% Moisture content of final blend	5.51	4.77	5.66	3.42	—
Weight variation (g)	ND	ND	0.96 ± 0.01	1.00 ± 0.02	1.0 g ± 5%
Thickness (mm)	ND	ND	5.44 ± 0.05	6.16 ± 0.14	—
Hardness (kp)	ND	ND	9.78 ± 0.31	9.60 ± 1.27	8–12 kp
Friability (%)	ND	ND	0.90	0.04	Not more than 1.0%
Disintegration time (minutes:seconds)	ND	ND	ND	20:25–34:48 (range, *n* = 6)	—

*Note:* Disintegration time is expressed as minutes: seconds (range of 6 determinations). All other values are presented as mean ± SD.

Abbreviation: ND, not determined.

**FIGURE 4:**
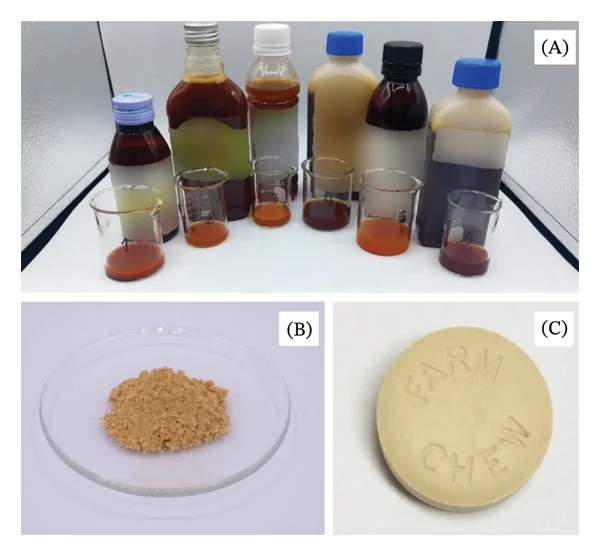
Various commercial products of the AC recipe in Thailand (A), AC‐SD powder (B), and the chewable tablet of AC‐SD (C).

A limitation of the present study is that individual tablet content uniformity was not evaluated. Given the high load (75% w/w) of spray‐dried botanical extract and the inherent heterogeneity of herbal materials, future studies should include content uniformity testing according to USP < 905 > and blend uniformity analysis to ensure consistent dosing of the active phytochemicals. Additionally, dissolution or in vitro release testing of the four marker compounds (GA, CMD, EUG, GZ) was not evaluated in the present study. While disintegration time serves as a preliminary indicator of tablet performance, future studies should include compound‐specific dissolution testing under simulated salivary and/or gastric conditions to confirm efficient release of the bioactive constituents from the tablet matrix. This is particularly important given the high proportion of isomalt and PVP K90 in the formulation, which may influence release kinetics.

The hardness acceptance range of 8–12 kp was selected based on the mechanical integrity of the tablets. During preliminary formulation trials, tablets with hardness below 8 kp exhibited friability above 1% and visible edge chipping during handling. Although the selected hardness range may be relatively high for a chewable dosage form, it was considered appropriate at this feasibility stage to ensure adequate tablet integrity during handling, packaging, and transportation. Further formulation optimization will be required to reduce tablet hardness while maintaining acceptable mechanical strength and friability. The disintegration time was evaluated as an auxiliary physicochemical parameter rather than a primary acceptance criterion. The relatively long disintegration times (20:25–34:48 min:sec) may reflect the strong tablet matrix formed during compression. Because the tablets are intended to be chewed before swallowing, the conventional disintegration test may not fully represent their behavior during actual administration.

A limitation of the present study is that organoleptic evaluation (e.g., taste, mouthfeel, aftertaste, and overall acceptability) was not performed. Although flavoring agents (menthol, camphor), a sweetener (sucralose), and isomalt were incorporated into the formulation (F4) to improve palatability, the acceptability of the resulting chewable tablet to patients has not been experimentally confirmed. Given that patient acceptability is a key motivation for the chewable dosage form, future studies should include a formal sensory evaluation using a human panel to assess taste, texture, and overall acceptability and to guide further optimization of flavoring and sweetening agents.

### 3.4. Comparison of Major Compounds in Chewable Tablets and Commercial Products of AC Recipe

The effects of production processes on the major bioactive compounds in AC products were analyzed by determining the amounts of GA, CMD, EUG, and GZ in six commercial liquid products (P1–P6), a self‐prepared liquid AC formulation (P7), and the developed chewable tablets (F4), calculated on a per‐dose basis. The appearance of the liquid AC products (P1–P7), AC‐SD powder, and the developed chewable tablets (F4) is shown in Figure 4A–C, respectively. The standard dose for liquid formulations was 30 mL, whereas one tablet constituted a single dose for the chewable formulation. The amounts of the major compounds varied considerably among the commercial products, ranging from 0.00 to 3.30 mg for GA, 0.14 to 0.53 mg for CMD, 2.03 to 20.38 mg for EUG, and 1.96 to 6.06 mg for GZ (Table 7). This variability may reflect differences in raw materials, processing conditions, and storage environments among the products.

**TABLE 7:** Major compounds in the commercial products of AC recipe, self‐prepared liquid form of the AC recipe, and developed chewable tablets.

Products	Dosage from	Shelf‐life	Volume/bottle (mL)	Color	Turbidity	Packaging	pH	Amount (mg/dose)^a^			
								GA	CMD	EUG	GZ
P1	Liquid	2 years	120	Russet	+++	Amber plastic bottle with aluminum cap	3.57	3.30 ± 0.10	0.49 ± 0.01	20.38 ± 0.86	1.58 ± 0.13
P2	Liquid	1 year	240	Russet	+	Clear plastic bottle with aluminum cap	4.42	2.21 ± 0.27	0.22 ± 0.02	15.73 ± 0.60	2.34 ± 0.06
P3	Liquid	1 year	180	Russet	−	Clear plastic bottle with plastic cap	4.91	0.35 ± 0.01	0.15 ± 0.01	12.17 ± 0.41	2.21 ± 0.05
P4	Liquid	1 year	240	Dark brown	+	Turbid plastic bottle with plastic cap	4.35	0.09 ± 0.01	0.30 ± 0.01	3.58 ± 0.12	1.96 ± 0.15
P5	Liquid	1 year	180	Russet	+	Amber plastic bottle with plastic cap	5.25	0.79 ± 0.01	0.53 ± 0.01	4.62 ± 0.20	6.06 ± 0.08
P6	Liquid	1 year	180	Dark brown	−	Turbid plastic bottle with plastic cap	4.46	0.00 ± 0.00	0.14 ± 0.01	2.03 ± 0.12	2.32 ± 0.11
P7	Liquid	—	180	Dark brown	+++	Clear plastic bottle with aluminum cap	4.34	10.38 ± 0.36	1.03 ± 0.02	8.22 ± 0.11	5.33 ± 0.10
F4	Tablets	—	—	Pale yellow	−	Aluminum foil	—	5.25 ± 0.06	0.36 ± 0.01	0.11 ± 0.01	1.74 ± 0.16

^a^P1–P7: 1 dose = 30 mL; F4: 1 dose = 1 tablet.

Products packaged in transparent or opaque plastic bottles (P2–P4 and P6) tended to show lower concentrations of CMD, which is reported to be sensitive to light exposure [41]. Similarly, products with aluminum caps (P1–P2) appeared to retain higher EUG levels than those with plastic caps. However, these observations were based on different commercial brands that also varied in raw material sourcing, manufacturing processes, and storage conditions. Therefore, the apparent association between packaging characteristics and compound content cannot be attributed solely to packaging materials. A controlled packaging‐exposure study using a single batch is recommended as a hypothesis‐driven future investigation to isolate the effects of packaging and cap materials on the stability of volatile bioactive compounds.

The developed chewable tablet showed lower amounts of several major compounds, particularly EUG, than the self‐prepared liquid formulation (P7). Compared with the commercial liquid formulations (P1–P6), F4 contained higher GA (5.25 mg/dose) than all commercial products (0.00–3.30 mg/dose), while CMD (0.36 mg/dose) was within the commercial range (0.14–0.53 mg/dose). In contrast, EUG levels in the commercial products (2.03–20.38 mg/dose) were approximately 18–185 times the level in F4 (0.11 mg/dose), and GZ (1.74 mg/dose) was lower in F4 than in five of the six commercial products (1.58–6.06 mg/dose), being higher only than P1 (1.58 mg/dose).

Chromatographic analysis (Figure 2B–D) showed a decreasing trend in CMD, EUG, and GZ during spray drying and subsequent tableting, whereas GA showed an apparent increase in the final tablet relative to AC‐SD. The detection of GA in the final tablet does not preclude partial degradation during spray drying, as residual GA may remain after thermal processing. Therefore, the apparent increase in the GA content from AC‐SD to the final tablet should not be interpreted as the formation of additional GA during tableting. Instead, it may reflect differences in the sample matrix, sample preparation, and extraction efficiency. Differences in the matrix of herbal tablet formulations may affect GA extraction and recovery and consequently influence the measured GA content [42]. Notably, the GA content in the final tablet (5.25 mg/dose) remained lower than that in the self‐prepared liquid formulation (10.38 mg/dose). As extraction recovery and matrix effects were not independently evaluated at each processing stage, the relative contributions of GA degradation and differences in extraction efficiency or matrix effects to the observed GA content cannot be confirmed. The reduction in GA during spray drying may be associated with thermal exposure at 120°C, the lowest temperature evaluated during preliminary screening. GA may undergo thermal degradation at elevated temperatures, including conversion to pyrogallol [43]. CMD and EUG are also susceptible to thermal loss through evaporation and oxidation, respectively [44–46], while GZ may undergo thermal hydrolysis [47]. Further optimization of spray‐drying conditions is therefore warranted to minimize the loss of thermolabile and volatile constituents. Regarding the safety of camphor, the F4 chewable tablet contained 1 mg of camphor per tablet, corresponding to a maximum daily exposure of 3 mg/day at the intended dosage of one tablet three times daily. According to the European Food Safety Authority (EFSA), a tolerable daily intake (TDI) for camphor could not be established because of limitations in the available toxicological data. However, EFSA considered a chronic exposure level of 15 mg/day (equivalent to 250 µg/kg body weight/day) as a conservative reference point and identified no safety concern regarding chronic toxicity at this exposure level. The estimated camphor exposure from the developed formulation (3 mg/day) is substantially below this level. Nevertheless, because camphor is intended for repeated oral administration in the present formulation and a formal TDI has not been established, its concentration should be appropriately controlled, and its safety should be further evaluated in subsequent product development.

## 4. Conclusion

This study demonstrated the feasibility of converting the traditional AC formulation into a chewable tablet using spray drying and wet granulation. MD‐18 at an extract‐to‐carrier ratio of 1:2 produced spray‐dried powder with suitable flowability, while isomalt served as an effective filler–binder for tablet production. The developed F4 tablet showed satisfactory physicochemical properties and practical advantages in handling and transportation. However, the manufacturing process resulted in compound‐specific losses of marker compounds, with reductions of approximately 99% in EUG, 35% in CMD, and 33% in GZ compared with the self‐prepared liquid formulation, while GA was relatively well preserved. Whether the chewable tablet provides improved physical stability relative to the liquid formulation remains to be confirmed through dedicated stability studies, and the chemical stability of volatile and heat‐sensitive constituents, particularly EUG, remains a critical challenge. Organoleptic acceptability was not evaluated and should be addressed in future sensory studies. The single‐batch design, absence of thermal characterization (e.g., DSC and FTIR), and lack of compound‐specific in vitro dissolution/release testing are additional limitations. Future studies should include independent replicate batches, thermal characterization, dissolution/release testing, and optimization of spray‐drying conditions or alternative approaches to minimize thermal degradation and improve product quality.

## Funding

The research was financially supported by the Thai traditional medical knowledge fund.

## Disclosure

The authors alone are responsible for the content and writing of this article.

## Ethics Statement

No approval by an ethics committee was required for the present study because no patient‐related data were used.

## Conflicts of Interest

The authors declare no conflicts of interest.

## Data Availability

The data supporting the findings of this study are available from the corresponding author upon reasonable request.
